# Association between dietary intakes of B vitamins and nonalcoholic fatty liver disease in postmenopausal women: a cross-sectional study

**DOI:** 10.3389/fnut.2023.1272321

**Published:** 2023-10-19

**Authors:** Jiajie Li, Jingda Huang, Yanqing Lv, Huifan Ji

**Affiliations:** ^1^Department of Hepatobiliary and Pancreatic Medicine, Infectious Disease. and Pathogen Biology Center, The First Hospital of Jilin University, Changchun, Jilin, China; ^2^Department of Nephrology, The First Hospital of Jilin University, Changchun, Jilin, China

**Keywords:** B vitamins, postmenopausal women, NAFLD, liver fibrosis, National Health and Nutrition Examination Survey

## Abstract

**Background:**

Non-alcoholic fatty liver disease (NAFLD) is increasingly common globally, particularly among postmenopausal women. Diet plays a fundamental role in the treatment of NAFLD. However, clinical research on the dietary intakes of B vitamins, specifically in postmenopausal women, is scant. Hence, it is imperative to study the impact of B vitamin dietary intake in postmenopausal women.

**Methods:**

This study utilized National Health and Nutrition Examination Survey (NHANES) data for 668 postmenopausal women. Logistic regression analysis was conducted to investigate the association of the intakes of B vitamins with hepatic steatosis and liver fibrosis prevalence. The analysis accounted for various covariates and employed restricted cubic spline analysis to examine potential nonlinear relationships. Additionally, interactions among age, diabetes, and B-vitamin intakes, as well as the interaction between folate and vitamin B12 intake, were explored.

**Results:**

Higher intakes of folate [0.30 (0.10–0.88)], choline [0.26 (0.07–0.95)], vitamin B1, and vitamin B2 were associated with a reduced risk of hepatic steatosis in postmenopausal women. The associations of niacin (P-nonlinear = 0.0003), vitamin B1 (P-nonlinear = 0.036), and vitamin B2 (P-nonlinear<0.0001) intakes with hepatic steatosis showed a nonlinear pattern. However, no significant associations were observed between the intakes of niacin, vitamin B6 and vitamin B12 and hepatic steatosis. Furthermore, there were no significant associations between B-vitamin intakes and liver fibrosis. No interaction effects were observed.

**Conclusion:**

Dietary intakes of folate, choline, vitamin B1, and vitamin B2 may be associated with liver steatosis in postmenopausal women, these results suggest that optimizing the intake of these specific B vitamins may have a protective effect against liver steatosis in postmenopausal women, offering valuable insights into potential dietary strategies to promote their well-being.

## 1. Introduction

In recent years, nonalcoholic fatty liver disease (NAFLD), now referred to as MASLD (Metabolic Dysfunction Associated Steatotic Liver Disease), has surpassed viral hepatitis as the most prevalent liver disease worldwide, with an estimated global prevalence of approximately 25%. It is further estimated that there are approximately 3.6 million new cases annually (1). Despite the renaming of NAFLD to MASLD, there was still controversy over the nomenclature when we conducted this study. Furthermore, the latest research has shown minimal differences between NAFLD and MASLD, with only one out of 414 liver biopsy cases of NAFLD not meeting the criteria for MASLD. Therefore, in the subsequent text, we will continue to use the term “NAFLD “(2).

While NAFLD is more prevalent in males (3), recent reports indicate an increasing trend in NAFLD prevalence among women (4). Notably, postmenopausal women, who lack estrogen, are susceptible to developing NAFLD and advanced NASH fibrosis, as compared to their premenopausal counterparts (5). Furthermore, a study indicates that for women, reaching the age of 50 or older significantly escalates the risk of advanced fibrosis (6). Given these observations, it becomes imperative to delve into the factors that contribute to or shield against NAFLD in postmenopausal women.

There is no specific medication available for NAFLD, and thus lifestyle modification remains the most effective long-term treatment, with dietary interventions being widely considered as the cornerston (7). Considering the significant influence of vitamins on the immune system and their potential to impact NAFLD, it’s crucial to investigate the role of dietary vitamin intake. While some studies have explored vitamins D, C, and E in NAFLD treatment (8, 9), there is a lack of studies on vitamin B complex and NAFLD, particularly in the context of postmenopausal women, with choline being the primary focus.

The vitamin B group encompasses eight water-soluble compounds, including thiamine (B1), riboflavin (B2), niacin (B3), pantothenic acid (B5), pyridoxine (B6), biotin (B7), folate (B9), and cyanocobalamin (B12). However, only a few of these compounds have been studied in relation to NAFLD. Vitamins B1 and B2, along with their active coenzymes thiamine pyrophosphate (TPP) and flavin adenine dinucleotide (FAD), respectively, play a crucial role in the catabolism (breakdown) of carbohydrates and fatty acids. Compared to premenopausal women, postmenopausal women are unable to synthesize choline (vitamin B4) through the endogenous pathway catalyzed by phosphatidylethanolamine N-methyltransferase (PEMT) due to the lack of estrogen (10, 11). This leads to significantly more severe fibrosis in postmenopausal women with low choline intake (12). Vitamin B3(niacin), serves as a precursor for the coenzyme nicotinamide adenine dinucleotide (NAD) and nicotinamide adenine dinucleotide phosphate (NADPH) and shows potential for the treatment of NAFLD (13). The relationship between vitamin B12, folate, and NAFLD has been a subject of ongoing debate due to the lack of direct clinical evidence for NAFLD treatment (14–17). However, in recent research conducted by Dr. Tripathi and colleagues, they investigated the effects of vitamin B12 and folate supplementation in dietary and Cbs knockdown (Cbs-LKD) mouse models, as well as in 36 patients, and primates. After adding folate and vitamin B12 to the drinking water from weeks 0 to 16, they observed histological improvements in liver cell infiltration of inflammatory cells and fibrosis in mice(except steatosis), suggesting the potential of dietary vitamin B12 and folate supplementation for the prevention or treatment of NASH (18). There is limited research on the other B vitamins and their relationship with NAFLD.

In light of this, our study aims to investigate the association between B vitamin intake (B1, B2, niacin, B6, folate, B12, and choline) and hepatic steatosis in postmenopausal women and assess their clinical correlation with liver fibrosis in postmenopausal women with NAFLD, utilizing data from the National Health and Nutrition Examination Survey (NHANES) 2017–2020 March.

## 2. Materials and methods

### 2.1. Study population

NHANES is a cross-sectional, complex, multistage survey conducted with a cycle of 2 years, aiming to evaluate the health and dietary condition of adults and children in the United States. The analysis utilized data from the NHANES cycles of March 2017–2020, as these studies included information related to liver steatosis and fibrosis detected using transient elastography in adult subjects. Due to the COVID-19 pandemic, data for 2020 was not completed and the collected data are not nationally representative. Therefore, the data from March 2019–2020 were combined with the cycles from 2017–2018.

The inclusion criteria for this study consisted of postmenopausal women between the ages of 18 and 80. Exclusion criteria were as follows: (1) participants with missing data on vitamin intake in their diet, red blood cell (RBC) folate levels; (2) presence of hepatitis B or C (defined by the detection of hepatitis C antibodies or the presence of hepatitis B surface antigen) or a history of liver cancer or autoimmune liver disease; (3) missing transient elastography data or failure to meet the criteria; and (4) excessive alcohol consumption (> 10 g/day). After excluding all participants who did not meet the criteria, a total of 668 postmenopausal women remained. Flowchart 1 illustrates the detailed screening process. The protocol received approval from the Institutional Review Board of the National Center for Health Statistics, and all participants provided written informed consent.

### 2.2. Dietary assessment

The dietary intake of B vitamins and cholesterol was assessed using a 24-h dietary recall method administered by trained interviewers on both weekdays and weekends. Two dietary assessments were conducted for each participant, with a time interval of 3 to 10 days between them. The first assessment was conducted face-to-face, while the second assessment was conducted *via* telephone. The energy, macronutrient, and micronutrient intake from the dietary recalls were analyzed. The intake of B vitamins and cholesterol from dietary supplements was also included in the study. The dietary data were collected using the automated multiple-pass method, and all food and beverage ingredients were adhered to standards set by the US Department of Agriculture Food and Nutrition Database. In this analysis, the dietary intake of B vitamins and cholesterol was evaluated using the single-day values for individuals with single recalls and the two-day average values for others. Due to the inability to establish a relationship between cholesterol intake exceeding 300 mg/day and cardiovascular diseases, the 2015–2020 Dietary Guidelines for Americans removed the restriction on recommending a maximum intake of 300 mg/day. However, those guidelines still advise minimizing the intake of cholesterol. Therefore, we defined cholesterol intake exceeding 300 mg/day as excessive.

### 2.3. Laboratory results

The detailed information regarding the measurement of RBC folate and urinary uric acid can be found in the laboratory method files on the NHANES official website (19).

### 2.4. Transient elastography

The main purpose of NHANES liver ultrasonographic transient elastography is to obtain objective measurements for two important manifestations of liver disease, liver fibrosis and hepatic steatosis, by recording controlled attenuation parameter (CAP), a method used to grade liver fat by measuring the degree of ultrasound attenuation in the liver) and FibroScan readings. The elastography examination is performed by NHANES health technicians who have received training and certification from NHANES staff and equipment manufacturers. Hepatic steatosis is characterized by a controlled attenuation parameter (CAP) score of 248 dB/m or above, while significant fibrosis is defined as a liver stiffness value equal to or exceeding 7.9 kPa (20, 21).

### 2.5. Other covariates

According to previous studies, we included the following risk factors for NAFLD as covariates: age (divided into an 18–60 years old group and a > 60 years old group), race/ethnicity (Mexican–American, Other Hispanic, Non-Hispanic White, Non-Hispanic Black, Other Races), education (≤ high school, >high school), daily physical activity (categorized based on the intensity of exercise and whether it is sustained for at least 10 min per week), smoking (current smoker, former smoker, never smoker), hypertension, diabetes, “overweight.group” (under/normal weight: <25 kg/m^2^, overweight: 25–30 kg/m^2^, obesity: ≥30 kg/m^2^). Menopausal women were defined as those who had not had a menstrual period in the previous 12 months because of hysterectomy, menopause or change of life. Diabetes was defined as follows: a previous diagnosis of diabetes or the use of diabetes medication for blood glucose control. If there was no previous diagnosis of diabetes, it was defined by a glycated hemoglobin (HbA1c) level of ≥6.5% or a fasting blood glucose level of ≥126 mg/dL. If the subjects were regularly taking antihypertensive medication, if their systolic blood pressure exceeded 130 millimeters of mercury, or if their average diastolic blood pressure exceeded 80 millimeters of mercury (based on the average of three measurements), they were diagnosed with hypertension.

### 2.6. Statistical analysis

After applying the recommended weighting from NHANES to all variables, we conducted logistic regression analysis to assess the relationships between B vitamin intakes and hepatic steatosis and liver fibrosis. Participants were categorized into four groups based on their B-vitamin intake quartiles, with Quartile 1 serving as the reference group. Odds ratios (ORs) and 95% confidence intervals were calculated to compare Quartile 2, Quartile 3, and Quartile 4 with the reference group regarding the presence of hepatic steatosis (fatty liver). The same methodology was applied to compare these groups among postmenopausal women with NAFLD (*n* = 435). Furthermore, we constructed three logistic regression models by progressively including covariates in the analysis. Model 1 was adjusted for age, overweight and race/ethnicity. In Model 2, we adjusted for education level, physical activity, and smoking, in addition to the covariates included in Model 1. In Model 3, we adjusted for diabetes, uric acid levels, hypertension, and dietary cholesterol intake, in addition to the covariates included in Model 2. To ensure robustness of the regression model, we employed the variance inflation factor (VIF), which assesses the level of high correlation among independent variables in multiple regression analysis, to examine the presence of multicollinearity in the logistic regression model. In cases where high multicollinearity was detected, we removed the covariate with the highest VIF value. In Model 3 of the study investigating the relationships of B-vitamin intakes with hepatic steatosis, the covariate “overweight.group” was removed due to multicollinearity. In the study on the relationships of B-vitamin intakes with liver fibrosis, “race” was excluded in Model 2 due to multicollinearity, and both “race” and “smoking” were excluded as covariates in Model 3. We also conducted a stratified analysis based on ethnicity (including only non-Hispanic White, non-Hispanic Black, and Mexican American individuals) with a sample size of 334. Additionally, we conducted restricted cubic spline analyses to examine whether there were nonlinear relationships in the model (with four knots at 5, 35, 65, and 95%). If a nonlinear relationship was found to be statistically significant (*p* < 0.05), we illustrated the relationship between vitamin intake and the dependent variable using graphical plots. We also investigated the interactions between age (treated as a categorical variable) and B-vitamin intakes using the multiplication interaction approach. All statistical analyses were carried out utilizing R version 4.1.2, with a significance threshold set at *p* ≤ 0.05 to establish statistical significance.

## 3. Results

### 3.1. Population characteristics

There were a total of 15,560 participants from March 2017 to 2020, with 2,208 of them being postmenopausal women. According to the exclusion criteria, we initially removed participants who lacked B-vitamin intake data (n = 129) and/or had insufficient or noncompliant transient elastography data (n = 248). Then, we excluded patients who might have had other liver diseases (*n* = 259), such as those with hepatitis B and C and those with excessive alcohol consumption, and those with missing covariate data (n = 784). Finally, a total of 668 subjects met the criteria for inclusion in this analysis. As shown in Table 1, the weighted prevalence of liver steatosis was 65.11%, while among patients with NAFLD, the weighted prevalence of liver fibrosis was 17.70%. In postmenopausal women, the prevalence of diabetes, obesity, hypertension, and hyperuricemia was higher among women with hepatic steatosis. Patients with hepatic steatosis (fatty liver) had lower intake of folate, choline, niacin, and vitamin B1. However, no such differences were observed in patients with liver fibrosis.

**Table 1:** Population characteristics by presence of non-alcoholic fatty liver disease and liver fibrosis in postmenopausal women.

		NAFLD group			Liver fibrosis group		
Characteristic	Overall, N = 668 (100%)^a^	NO, N = 233 (34.8%)^a^	YES, N = 435 (65.2%)a	*p*-value^b^	NO, N = 358 (82.3%)^a^	YES, N = 77 (17.7%)^a^	*p*-value^b^
**age.group**				0.8			0.8
18-60 years	255 (38.2%)	95 (40.8%)	160 (36.8%)		128 (35.8%)	32 (46%)	
60 + years	413 (61.8%)	138 (59.2%)	275 (63.2%)		230 (64.2%)	45 (54%)	
**Weight.group**				<0.001			0.025
Normal	142 (21.3%)	91 (39.1%)	51 (11.8%)		47 (13.1%)	4 (4.0%)	
Obesity	335 (50.1%)	61 (26.2%)	274 (63.0%)		211 (58.9%)	63 (84%)	
Overweight	191 (28.6%)	81 (34.8%)	110 (25.3%)		100 (27.9%)	10 (NA%)	
**Race**				0.5			0.5
Mexican American	60 (9.0%)	15 (6.4%)	45 (10.3%)		33 (9.2%)	12 (8.6%)	
Other Hispanic	64 (9.5%)	23 (9.9%)	41 (9.4%)		35 (9.8%)	6 (3.8%)	
Non-Hispanic White	264 (39.5%)	93 (39.9%)	171 (39.3%)		142 (39.7%)	29 (64%)	
Non-Hispanic Black	179 (36.8%)	61 (26.2%)	118 (27.1%)		94 (26.3%)	24 (12%)	
Others/multiracial	101 (15.1%)	41 (17.5%)	60 (13.8%)		54 (12%)	6 (11%)	
**Smoke**				0.2			0.2
Current	92 (13.8%)	36 (15.5%)	56(12.9%)		52 (14.5%)	4 (5.2%)	
Former	149 (21.4%)	45 (19.3%)	104 (23.9%)		87 (24.3%)	17 (22.1%)	
Never	426 (63.8%)	152 (65.2%)	275(63.2%)		219 (61.2%)	56 (72.7%)	
**Hypertention**	467 (69.9%)	147 (63.1%)	320 (73.6%)	0.002	257 (71.8%)	63 (86%)	0.002
**Hyperuricemia**	152 (22.8%)	32 (13.7%)	120 (27.6%)	0.019	92 (25.6%)	28 (36%)	0.087
**Diabetes**	188 (28.1%)	31 (13.3%)	157 (36.1%)	<0.001	114 (31.8%)	43 (63%)	<0.001
**Exercise**				0.009			0.089
No exercises	388 (58.1%)	127 (54.5%)	261 (60.0%)		209 (58.4%)	52 (67.5%)	
Moderate exercises	202 (30.2%)	68 (29.2%)	134 (30.8%)		111 (31.0%)	23 (29.9%)	
Vigorous exercises	78 (11.7%)	38 (16.3%)	40 (9.2%)		38 (10.6%)	2 (2.6%)	
**Education**				0.7			0.4
<=High school	284 (42.5%)	93 (39.9%)	191 (43.9%)		155 (43.3%)	36 (46.8%)	
>High school	384 (57.5%)	140 (60.1%)	244 (56.1%)		203 (56.7%)	41 (53.2%)	
**Cholesterol(mg/d)**				0.4			0.007
excessive	223 (33.4%)	66 (28.3%)	157 (36.1%)		122 (34.1%)	35 (45.5%)	
suitable	445 (66.6%)	167 (71.7%)	278 (63.9%)		236 (65.9%)	42 (54.5%)	
**Choline(mcg/d)**	272.54 (131.16)	309.92 (124.96)	227.51 (124.37)	0.10	256.52 (118.13)	354.30 (195.29)	0.012
**Folate(mcg/d)**	526.12 (397.09)	666.27 (469.24)	452.19 (330.56)	0.001	457.32 (334.37)	425.52 (310.70)	0.6
**Niacin(mg/d)**	34.30 (69.30)	32.67 (20.50)	35.16 (84.37)	0.023	36.30 (90.77)	29.21 (35.66)	>0.9
**Vitamin 6(mg/d)**	5.96 (19.33)	9.73 (28.64)	3.97 (11.30)	0.008	3.44 (5.22)	6.74 (25.48)	0.5
**Vitamin 12(mcg/d)**	89.40 (264.43)	60.01 (199.06)	124.82 (323.02)	0.2	137.32 (336.70)	38.25 (184.33)	0.12
**Vitamin 1(mg/d)**	2.27 (4.02)	2.94 (6.36)	1.95 (2.03)	0.3	1.93 (2.17)	2.04 (1.06)	0.2
**Vitamin 2(mg/d)**	5.69 (18.00)	9.18 (24.37)	3.85 (13.15)	0.005	3.86 (13.26)	3.78 (12.63)	0.5
**RBC Folate(nmol/L)**	1,353.71 (568.00)	1,368.74 (516.89)	1,345.79 (593.61)	0.3	1,345.87 (598.39)	1,345.37 (571.99)	>0.9

a *n* (unweighted) (%); Mean (SD).

b chi-squared test with Rao & Scott’s second-order correction; Wilcoxon rank-sum test for complex survey samples.

NAFLD: non-alcoholic fatty liver disease.

### 3.2. Associations with liver steatosis

In general, the results regarding the associations of dietary B-vitamin intakes with hepatic steatosis in postmenopausal women were similar between Model 1 and Model 2. Additionally, Model 3 showed similar results to Model 1 and Model 2, except for the associations involving the following B vitamins: folate, vitamin B6, choline, and niacin. In Model 2, comparing the highest and lowest B-vitamin intake levels, a higher intake of vitamin B6 [OR (95% CI): 0.27 (0.10–0.74)] was negatively associated with hepatic steatosis. However, in Model 3, a higher intake of vitamin B6 was only weakly negatively associated with hepatic steatosis [0.33 (0.11–1.02)]. Similar to previous findings, in all three models, the highest intake level of folate was negatively associated with hepatic steatosis when compared with the lowest intake level [Model 3: 0.30 (0.10–0.88)]. This suggests that a higher intake of folate is a protective factor against NAFLD in postmenopausal women. However, no similar results were observed for RBC folate levels compared with those of dietary folate intake. For choline, we did not find statistically significant results in Models 1 or 2. However, in Model 3, after further adjustment for covariates, including diabetes, hypertension, cholesterol intake, and hyperuricemia, and the exclusion of obesity as a covariate due to collinearity, a higher intake of choline was shown to have a statistically significant negative association with hepatic steatosis [0.26 (0.07–0.95)]. Based on this result, we continued our research by gradually adding the aforementioned four covariates to Model 2, and it was found that including the covariate of cholesterol intake resulted in the aforementioned results. In addition, we found a negative association between a higher niacin intake and hepatic steatosis in Model 1. However, this relationship disappeared in Models 2 and 3. No links were observed between liver steatosis and other vitamins (see Table 2).

**Table 2:** Associations between B vitamin and non-alcoholic fatty liver disease in postmenopausal women.

Exposure	Model 1			Model 2			Model 3		
	Q2 vs. Q1 OR (95%CI)	Q3 vs. Q1 OR (95%CI)	Q4 vs. Q1 OR (95%CI)	Q2 vs. Q1 OR (95%CI)	Q3 vs. Q1 OR (95%CI)	Q4 vs. Q1 OR (95%CI)	Q2 vs. Q1 OR (95%CI)	Q3 vs. Q1 OR (95%CI)	Q4 vs. Q1 OR (95%CI)
Vitamin B1	0.76(0.34–1.68)	0.71(0.34–1.50)	0.46(0.18–1.13)	0.78(0.34–1.80)	0.70(0.30–1.80)	0.48(0.19–1.24)	0.60(0.22–1.63)	0.60(0.19–1.86)	0.47(0.16–1.39)
Vitamin B2	0.79(0.38–1.64)	0.81(0.31–2.10)	0.51(0.26–1.00)	0.76(0.35–1.66)	0.79(0.32–1.96)	0.52(0.26–1.01)	0.58(0.27–1.25)	0.61(0.23–1.66)	0.53(0.24–1.16)
Vitamin B6	0.88(0.42–1.83)	0.87(0.1–1.86)	0.26(0.10–0.70)*	0.91(0.41–1.22)	0.85(0.39–1.85)	0.27(0.10–0.74)*	0.92(0.38–2.22)	0.77(0.30–1.97)	0.33(0.11–1.02)
Vitamin B12	1.15(0.46–2.84)	0.59(0.22–1.57)	0.64(0.40–1.03)	1.23(0.47–3.23)	0.60(0.22–1.65)	0.67(0.41–1.09)	1.02(0.41–2.55)	0.62(0.19–1.97)	0.69(0.41–1.16)
Choline	0.85(0.30–2.42)	1.13(0.51–2.53)	0.74(0.34–1.61)	0.85(0.30–2.46)	1.22(0.50–3.01)	0.70(0.29–1.71)	0.61(0.21–1.77)	0.79(0.25–2.53)	0.26(0.07–0.95)*
Folate, DFE	1.35(0.54–3.37)	0.80(0.33–1.95)	0.28(0.10–0.78)*	1.48(0.53–4.17)	0.80(0.31–2.11)	0.30(0.10–0.89)*	0.93(0.38–2.27)	0.62(0.19–2.0)	0.30(0.10–0.88)*
Niacin	1.17(0.50–2.72)	0.94(0.40–2.18)	0.42(0.17–0.99)*	1.24(0.51–2.97)	0.94(0.38–2.31)	0.45(0.18–1.13)	0.98(0.38–2.54)	0.77(0.28–2.12)	0.48(0.17–1.36)
RBC folate	0.68(0.28–1.66)	0.56(0.21–1.46)	0.58(0.23–1.43)	0.72(0.27–1.89)	0.63(0.23–1.71)	0.60(0.24–1.51)	0.49(0.21–1.18)	0.59(0.23–1.49)	0.56(0.19–1.65)

Q1, quantiles 1; Q2, quantiles 2; Q3, quantiles 3; Q4 quantiles 4.

OR (95% CI), odds ratio (95% confidence interval).

*P < 0.05, **p < 0.01.

DFE, dietary folate equivalents.

Model 1 was adjusted for age.group, weight.group, and race/ethnicity.

Model 2 was adjusted for covariates in model 1, and also education, physical activity, smoking.

Model 3 was adjusted for covariates in model 2, and also hypertension, diabetes, and dietary intakes of cholesterol and hyperuricemia, while eliminate weight.group because of collinearity.

### 3.3. Associations with liver fibrosis

In patients with NAFLD, when comparing the intakes of the different B vitamins using quartiles, there was a significant variation in intakes between Model 3 and Models 1 and 2 (Supplementary Table S1). This may be due to the limited number of patients with liver fibrosis (*n* = 77) and the extremely uneven distribution of liver fibrosis patients among the four quartile groups. We also conducted a baseline analysis on four groups categorized by quartiles of B-vitamin intake, and we found no significant differences in their baseline characteristics (refer to Supplementary Table S3). Therefore, in this logistic regression model, we adopted the third quartile (Table 3) for analysis. Similar to the regression model for hepatic steatosis, the results of the liver fibrosis regression models in Models 1 and 2 showed fluctuations but no significant changes. Comparing the highest tertile to the lowest tertile in Model 2, a higher dietary intake of choline showed a positive association with liver fibrosis, with an odds ratio of 4.05 (95% confidence interval: 1.24–13.2). However, In Model 3, the aforementioned association became nonsignificant. There were no significant associations observed between intakes of the other B vitamins and liver fibrosis (Table 3). Due to the potential associations of race with dietary and genetic backgrounds, we also conducted a stratified analysis based on race (refer to Supplementary Table S2). In this analysis, we only included non-Hispanic White, non-Hispanic Black, and Mexican American individuals, resulting in a sample size of 334. The results showed significant fluctuations compared to those in Table 3; however, the overall trend remained consistent. In this stratified analysis, we did not observe any significant relationship between the intakes of B vitamins and liver fibrosis.

**Table 3:** Associations between B vitamin and liver fibrosis in postmenopausal women.

**Exposure**	Model 1		Model 2		Model 3	
	**T2 vs. T1 OR (95%CI)**	**T3 vs. T1 OR (95%CI)**	**T2 vs. T1 OR (95%CI)**	**T3 vs. T1 OR (95%CI)**	**T2 vs. Q1 OR (95%CI)**	**T3 vs. T1 OR (95%CI)**
Vitamin B1	1.58(0.71–3.52)	0.82(0.33–2.04)	1.89(0.82–4.37)	0.85(0.42–1.71)	1.84(0.86–3.93)	0.87(0.35–2.15)
Vitamin B2	1.61(0.54–4.85)	1.50(0.51–4.43)	1.96(0.59–6.49)	1.74(0.82–3.67)	1.70(0.57–5.05)	1.47(0.57–3.82)
Vitamin B6	1.06(0.33–3.40)	1.45(0.53–4.01)	1.14(0.32–4.03)	1.69(0.72–3.94)	1.05(0.34–3.28)	1.57(0.62–3.95)
Vitamin B12	1.36(0.50–3.72)	1.56(0.40–6.04)	1.23(0.37–4.07)	1.38(0.52–3.66)	1.03(0.37–2.89)	1.01(0.41–2.46)
Choline	2.47(0.76–8.05)	4.32(1.43–13.1)*	2.25(0.90–5.65)	4.05(1.24–13.2)*	1.69(0.45–6.40)	1.82(0.32–10.3)
Folate, DFE	1.09(0.39–3.01)	0.83(0.33–2.13)	1.21(0.50–2.96)	0.90(0.35–2.33)	1.04(0.39–2.81)	0.85(0.34–2.12)
Niacin	2.28(0.90–5.75)	1.04(0.28–3.82)	2.31(0.92–5.81)	1.09(0.40–2.98)	1.79(0.80–4.02)	0.80(0.26–2.45)
RBC folate	0.97(0.59–1.58)	0.97(0.63–1.49)	0.98(0.59–1.61)	0.98(0.62–1.53)	0.97(0.57–1.65)	0.97(0.59–1.59)

T1, tertiles 1; T2, tertiles 2; T3, tertiles 3.

OR (95% CI), odds ratio (95% confidence interval).

*P < 0.05, **P < 0.01.

DFE, dietary folate equivalents.

Model 1 was adjusted for age.group, weight.group, and race/ethnicity.

Model 2 was adjusted for covariates in model 1, and also education, physical activity, smoke, while eliminate race because of collinearity.

Model 3 was adjusted for covariates in model 2, and also hypertension, diabetes, and dietary intakes of cholesterol and hyperuricemia, while eliminate smoke because of collinearity.

### 3.4. Nonlinear relationships

**Figure 1:**
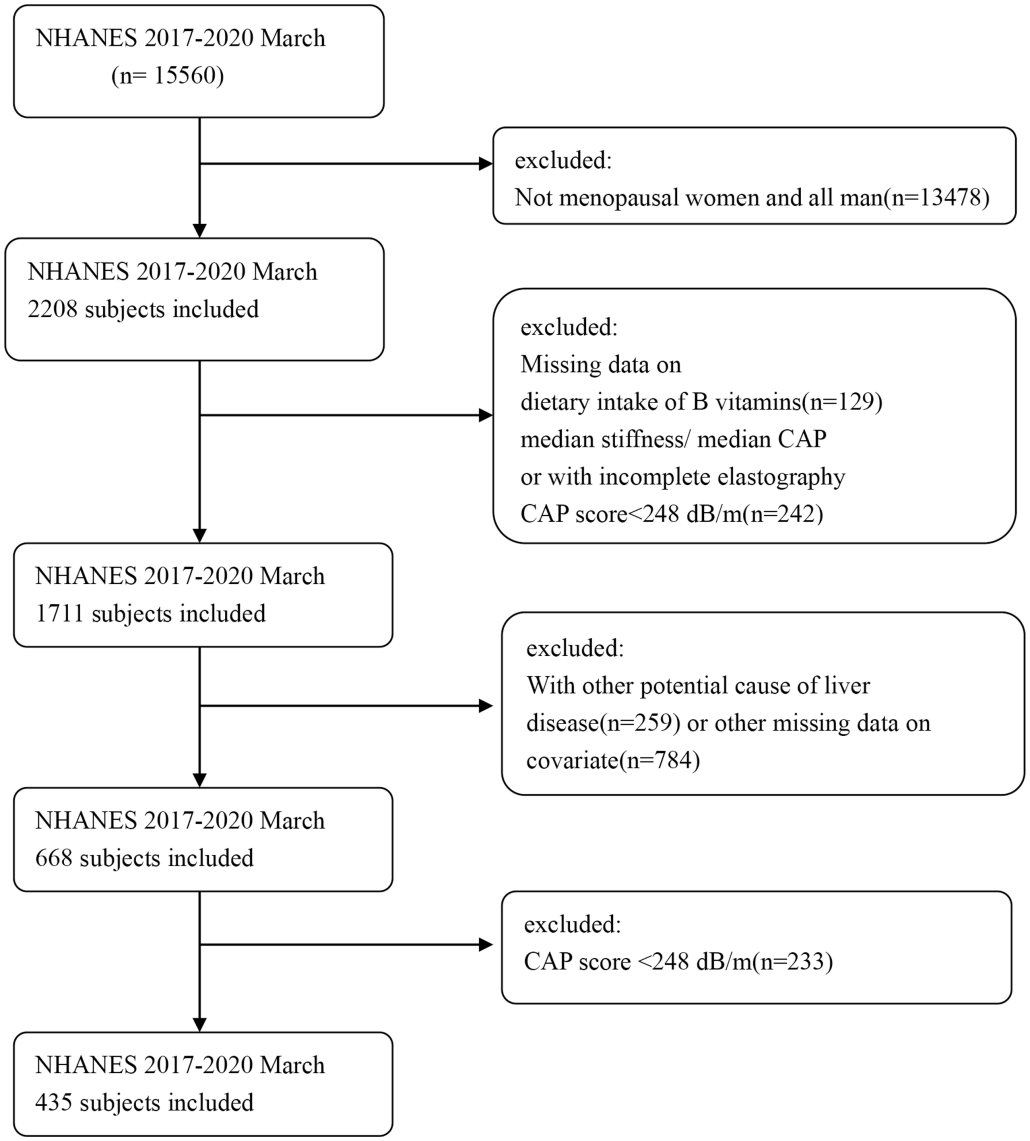
Flowchart of the study population.

**Figure 2:**
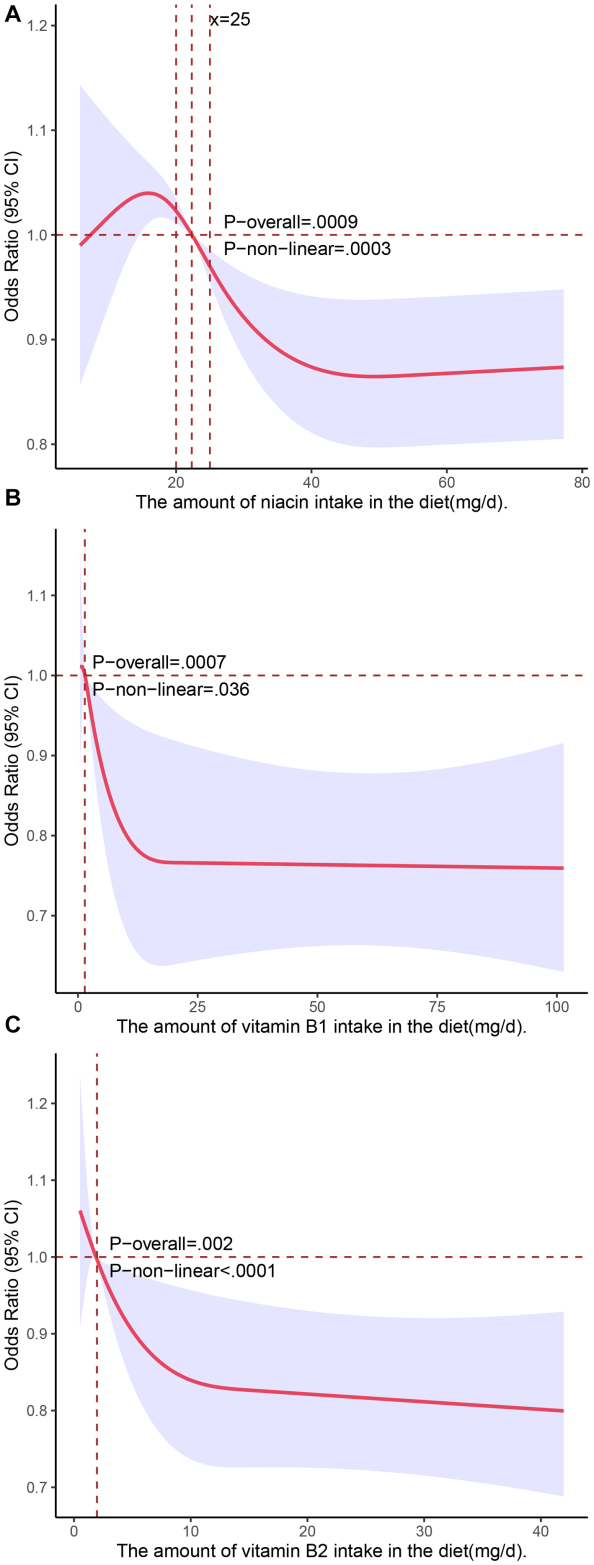
Associations of dietary intakes of niacin **(A)**, vitamin B1 **(B)**, and vitamin B2 **(C)** with the risk of NAFLD (presented as odds ratios) in postmenopausal women using a restricted cubic spline regression model.

We used restricted cubic splines in all of the Model 3 analyses to test for nonlinear relationships between the independent variables and the response variable and found that dietary intakes of vitamin B1, vitamin B2, and niacin exhibited nonlinear relationships with NAFLD. As shown in Figure 1, there was an inverted U-shaped association between niacin and NAFLD in menopausal females. When niacin intake was 15.8 mg, the odds ratio (OR) reached its highest point of 1.04 (95% confidence interval: 1.02–1.07). Subsequently, as the intake of niacin increased, the prevalence of NAFLD decreased. When the niacin intake was approximately 40 mg, the prevalence of NAFLD reached a plateau. We also investigated the relationship between niacin intake and NAFLD in the general population during the NHANES cycle from 2017 to March 2020 (*n* = 3,088) using restricted cubic splines, and the results revealed that there was no significant association between niacin intake and the prevalence of NAFLD (Supplementary Figure S2A). The associations of the dietary intakes of vitamin B2 and vitamin B1 with NAFLD followed a decreasing curve (Figures 2B,C). As shown in Figure 2B, a decrease in the risk of developing NAFLD was observed with an increasing intake of vitamin B1 when it exceeded 1.42 mg, and once the dietary intake of vitamin B1 exceeded 15 mg, the change in OR with a further increased intake became negligible. In Figure 2C, when vitamin B2 was greater than 1.75 mg (OR = 1), the risk of developing NAFLD decreased with the increasing intake of vitamin B2. When the dietary intake of vitamin B2 exceeded 10 mg, further increases in the intake resulted in minimal changes in the OR. These findings indicated that the protective effect against NAFLD does not continue to improve beyond dietary niacin intake of 40 mg, dietary vitamin B1 intake of 15 mg, and dietary vitamin B2 intake of 10 mg. We did not observe nonlinear relationships in other models.

### 3.5. The interaction effect in the model

In order to ensure the robustness of the results, we applied the Rao-Scott chi-square test or the likelihood ratio to explore the multiplicative interaction of age group and diabetes with B-vitamin intakes for all three models. The results indicated no significant multiplicative interaction between these variables. Additionally, we included the interaction term between dietary intake of vitamin B12 and folate in Models 1 and 3 to investigate whether there was a multiplicative interaction effect on NAFLD. Notably, due to the inclusion of the interaction term with quartiles, the model degrees of freedom were limited. Therefore, we categorized the intake of folate and vitamin B12 into tertiles instead of quartiles. As shown in Table 4, the *p* value for interaction was not statistically significant, but when we applied the Wald t test, the result was statistically significant (*p* = 0.012). The difference in results between the Wald t test and the Rao-Scott chi-square test can be attributed to their underlying assumptions and methodologies. The Wald t test assumes a normal distribution and is suitable for simple random samples. However, in the case of the NHANES study, which is based on complex survey sampling, the Rao-Scott chi-square test is more appropriate. This test accounts for the complex sampling design and uses weighted estimates to provide valid statistical inference. We also presented the results of the stratified interaction effect in the Table 4.

**Table 4:** Combined effect of dietary intake of folate and vitamin B12 on NAFLD.

Variable 1	Variable 2		
Folate intake	Vitamin B12 intake	Adjusted OR (95%Cl)	P for interaction
T1	T1	1.00(reference)	0.192
T1	T2	0.84(0.18–3.85)	
T1	T3	9.25(0.72–119)	
T2	T1	0.91(0.20–4.10)	
T2	T2	1.58(0.25–10.1)	
T2	T3	1.20(0.02–23.1)	
T3	T1	0.57(0.07–4.88)	
T3	T2	0.13(0.01–2.65)	
T3	T3	0.07(0.00–1.13)	

## 4. Discussion

The results of this nationwide survey among postmenopausal women suggest a negative potential association between the dietary intakes of folate, choline and vitamin B6 and hepatic steatosis. Additionally, restricted cubic spline analysis indicated a nonlinear relationship between vitamin B1, vitamin B2, niacin, and hepatic steatosis. In addition, there was no evidence of an interaction between age grouping and dietary intake of B vitamins, diabetes and dietary intake of B vitamins, or dietary intake of vitamin B12 and folate.

Several mouse models related to choline have demonstrated the association between choline-deficient diets and NAFLD (22, 23). These diets can disrupt the gut microbiota by reducing diversity and altering the microbial community, potentially activating the Nod-like receptor protein 3 (NLRP3) inflammasome (24). Additionally, choline-deficient diets can impair the intestinal barrier, allowing increased bacterial penetration from the gastrointestinal tract into organs like the liver, leading to hepatic steatosis and lipid accumulation (25, 26). The accumulation of lipids may be linked to abnormal fatty acid metabolism, inflammatory responses, and activation of the NLRP3 inflammasome. These research findings demonstrate the impact influence of insufficient choline on the progression of NAFLD. Cholesterol and choline were often found together in the same food. In our study, after adjusting for the effects of cholesterol, the relationship between choline and NAFLD became significant. In a recent study, it was found that an increased intake of eggs and choline in elderly women in the United States was significantly associated with an increased risk of diabetes. However, when cholesterol consumption was accounted for and adjusted for, the aforementioned relationship disappeared (27). This suggests that cholesterol intake may mask the relationship between choline and NAFLD. However, further research is needed to better understand these relationships and optimize dietary intakes of these nutrients to reduce the risk of this disease.

Folate plays a role in methylation processes, and folate deficiency has been linked to triglyceride (TG) accumulation in the liver and a lower risk of NAFLD (OR: 0.75; 95% CI: 0.58, 0.99) (15, 28). Additionally, dietary folate deficiency can lead to reduced levels of choline and phosphatidylcholine (PC) in the liver (29). As mentioned above, in a recent study, dietary folate was reported to promote the conversion of homocysteine to methionine, stimulate fatty acid β-oxidation, and improve liver histology in mouse models (18). However, the relationship between folate intake and NAFLD risk remains a topic of debate, as some studies have not observed an association with folate intake (30, 31). The Institute of Medicine (IOM) recommends a Recommended Dietary Allowance (RDA) of 400 micrograms of folate for adults aged 18 and older, with a Tolerable Upper Intake Level (UL) of 1,000 micrograms (32). In our study, we observed that higher folate concentrations (>698 mcg) in the Q4 group were negatively associated with the incidence of NAFLD compared to the Q1 group. The aforementioned findings suggest that folate supplementation in postmenopausal women may have the potential to reduce the risk of developing NAFLD. However, unmetabolized folic acid has been reported to reduce the cytotoxicity of natural killer cells, thereby compromising the immune system in the human body (33). Therefore, the intake of folate in elderly individuals needs to be considered comprehensively. In contrast to previous studies, our research did not find a relationship between RBC folate levels and NAFLD. This discrepancy may be because only one participant among the included postmenopausal women had a lower plasma RBC folate concentration.

Preclinical studies have shown that niacin inhibits the activity of hepatic cell DGAT2 and NADPH oxidase, leading to a reduction in hepatic lipid accumulation and ROS generation in the liver (34, 35). In an uncontrolled clinical trial involving 39 patients with hypertriglyceridemia and concurrent hepatic steatosis, treatment with extended-release niacin (ER) for 6 months resulted in a significant 47% reduction in liver fat (13). Nevertheless, it should be noted that there is still a lack of sufficient clinical evidence in this area. However, in our study, a protective effect was observed when the intake of niacin exceeded 22.3 mg (Figure 2A), which contributes to the clinical evidence supporting the above notion to some extent.

The mechanisms of vitamin B1 and vitamin B2 in NAFLD are not fully understood, and there is limited clinical research available on this topic. However, related animal experiments have revealed the potential of vitamin B1 supplementation in preventing NAFLD. In its active form thiamine pyrophosphate (TPP), thiamine (vitamin B1) may enhance carbohydrate metabolism by maximizing the activity of TPP-dependent enzymes, potentially reducing the rate of hepatic lipid accumulation. Recent animal experiments have demonstrated that thiamine supplementation can markedly reduce hepatic fat content in animal models consuming a high-calorie diet (36). In our study, we found nonlinear relationships of dietary intakes of vitamin B1 and vitamin B2 with NAFLD. Specifically, higher intakes of vitamin B1 and vitamin B2 were negatively associated with the risk of NAFLD. Consequently, providing adequate levels of Vitamin B1 and Vitamin B2 in the daily diets of postmenopausal women could potentially serve as a preventive measure against NAFLD. However, more clinical studies are needed to confirm and further understand this phenomenon.

Our study focused on postmenopausal women in the United States, which is a relatively understudied population. This study provides valuable information for understanding the relationship between dietary intake of B vitamins and NAFLD in postmenopausal women. Moreover, we found nonlinear associations of the intakes of niacin, vitamin B1 and vitamin B2 with NAFLD, with higher intakes being negatively associated with the risk of NAFLD. These findings offer new insights into the potential role of niacin, vitamin B1, vitamin B2, folate and choline in the prevention of NAFLD and can contribute to guiding dietary recommendations and strategies for NAFLD prevention in postmenopausal women, with practical implications for public health and clinical practice. However, there are some limitations to consider. First, our study was cross-sectional, and since the dietary data was collected for only 1–2 days, and due to limitations in data and software, we did not apply the NCI model or the SPADE model for dietary intake estimation, which could potentially introduce bias and might not fully represent the long-term dietary habits of the participants. Nonetheless, dietary data collection is inherently challenging in dietary research. Additionally, the observational nature of the study limits our ability to establish causality, which would require prospective studies for confirmation. Second, importantly, our study had a relatively small sample size, and the grouping based on quartiles may have resulted in a highly imbalanced distribution of the liver fibrosis (*n* = 77) across each group, potentially leading to erroneous results. However, we validated the results by testing different grouping methods (e.g., using tertiles) to minimize such errors. Last, our assessment of hepatic steatosis relied on ultrasound imaging rather than the gold standard of liver biopsy.

In conclusion, the findings of this study targeting postmenopausal women suggest that dietary intakes of folate, choline, vitamin B1, vitamin B2, and niacin may be negatively associated with NAFLD in this population. Specifically, folate and choline intakes showed a linear relationship with NAFLD, while the associations of vitamin B1, vitamin B2, and niacin with hepatic steatosis may be nonlinear. However, further prospective research is needed to confirm these observations and establish a clearer understanding of the relationships between these nutrients and NAFLD in postmenopausal women.

## Data availability statement

The original contributions presented in the study are included in the article/Supplementary material, further inquiries can be directed to the corresponding author.

## Ethics statement

The studies involving humans were approved by the National Center for Health Statistics Research Ethics Review Board. The studies were conducted in accordance with the local legislation and institutional requirements. The participants provided their written informed consent to participate in this study.

## Author contributions

JL: Conceptualization, Methodology, Writing – original draft, Writing – review & editing. JH: Supervision, Writing – original draft, Formal analysis. YL: Writing – original draft, Validation, Writing – review & editing. HJ: Supervision, Conceptualization, Writing – review & editing.
