# Supplementary figures and images for: Association between dietary intakes of B vitamins and nonalcoholic fatty liver disease in postmenopausal women: a cross-sectional study
Source: Front Nutr. 2023 Oct 19;10:1272321. doi: 10.3389/fnut.2023.1272321 (PMC10621796; doi:10.3389/fnut.2023.1272321)

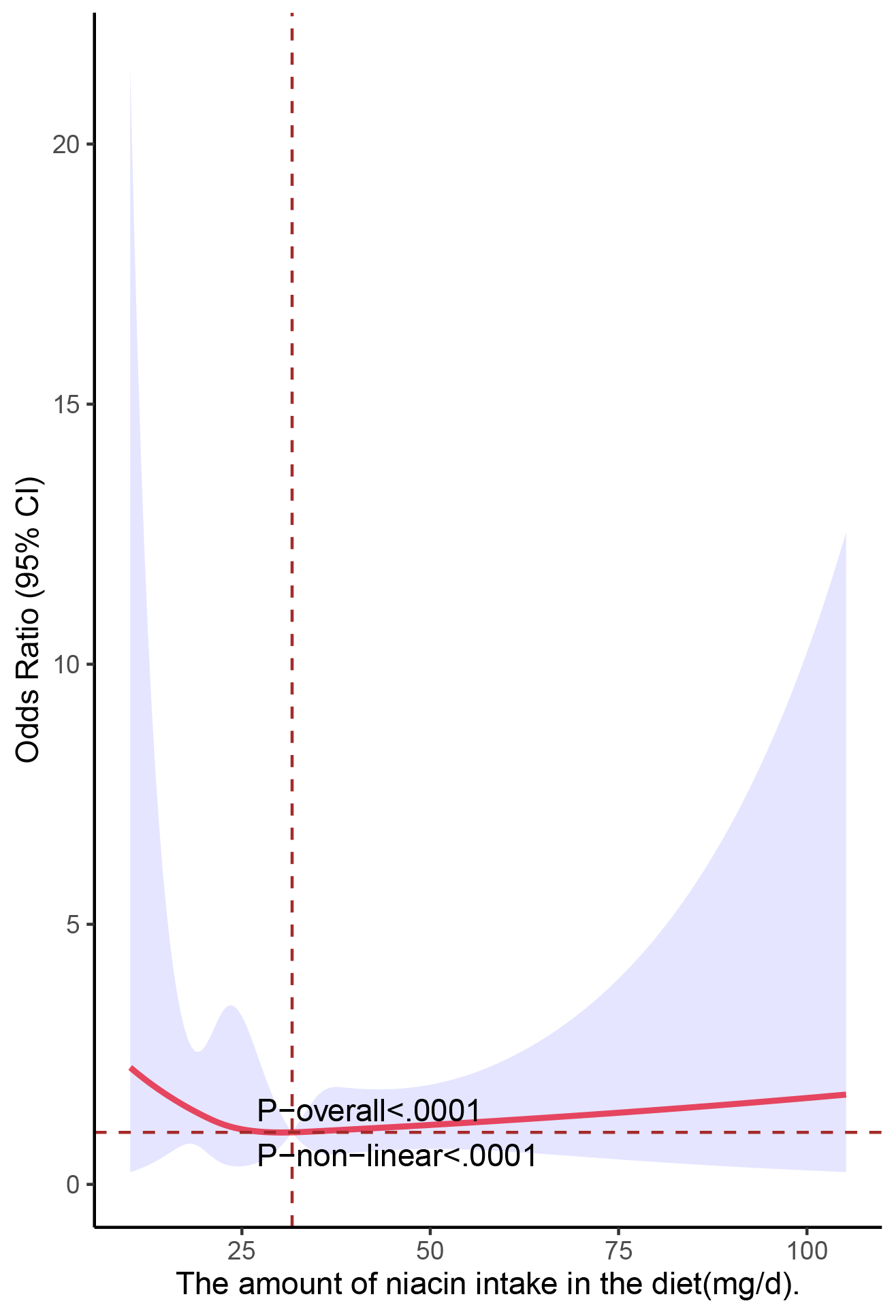

Supplement: Supplementary file 1 [file Image_1.TIF]
