# Supplementary material for: Association between dietary intakes of B vitamins and nonalcoholic fatty liver disease in postmenopausal women: a cross-sectional study
Source: Front Nutr. 2023 Oct 19;10:1272321. doi: 10.3389/fnut.2023.1272321 (PMC10621796; doi:10.3389/fnut.2023.1272321)
Supplement: Supplementary file 2 [file Table_1.DOCX]

**Supplementary table 1. Associations between B vitamin and liver fibrosis in postmenopausal women (quartile method)**

| **Exposure** | Model 1 Model 2 Model 3 | | | | | | | | |
| --- | --- | --- | --- | --- | --- | --- | --- | --- | --- |
|  | **Q2 vs. Q1**  **OR (95%CI)** | **Q3 vs. Q1**  **OR (95%CI)** | **Q4 vs. Q1**  **OR (95%CI)** | **Q2 vs. Q1**  **OR (95%CI)** | **Q3 vs. Q1**  **OR (95%CI)** | **Q4 vs. Q1**  **OR (95%CI)** | **Q2 vs. Q1**  **OR (95%CI）** | **Q3 vs. Q1**  **OR (95%CI)** | **Q4 vs. Q1**  **OR (95%CI)** |
| Vitamin B1 | 2.32(0.89-6.06) | 0.82(0.14-4.75) | 0.90(0.32-2.56) | 2.69(1.01-7.16)* | 0.80(0.17-3.80) | 1.04(0.45-2.38) | 3.72(1.62-8.54)** | 0.76(0.16-3.58) | 1.12(0.35-3.57) |
| Vitamin B2 | 5.48(1.98-15.2)** | 2.87(1.51-5.45)** | 3.93(1.24-12.4)* | 5.80(1.92-17.5)** | 2.77(1.27-6.04)* | 4.27(1.90-9.59)** | 5.05(1.58-16.2)* | 2.46(1.00-6.08) | 3.30(1.47-7.39)** |
| Vitamin B6 | 0.57(0.12-2.65) | 1.44(0.48-4.38) | 0.91(0.33-2.54) | 0.61(0.11-3.28) | 1.65(0.57-4.75) | 1.05(0.30-3.65) | 0.61(0.12-3.06) | 1.50(0.49-4.59) | 1.03(0.29-3.69) |
| Vitamin B12 | 2.61(1.13-6.01)* | 1.42(0.36-5.61) | 2.80(0.65-12.1) | 2.36(0.88-6.37) | 1.36(0.29-6.36) | 2.32(0.75-7.17) | 2.18(0.76-6.24) | 1.18(0.29-4.83) | 1.77(0.61-5.18) |
| Choline | 4.62(0.79-26.9) | 3.90(0.94-16.1) | 8.83(1.99-16.1)** | 4.98(1.19-20.9)* | 3.24(0.87-12.1) | 8.67(2.13-35.3)** | 4.02(0.73-22.3) | 1.69(0.48-6.71) | 2.52(0.49-12.9) |
| Folate, DFE | 2.00(0.63-6.31) | 1.32(0.53-3.25) | 0.73(0.23-2.33) | 1.99(0.66-5.75) | 1.22(0.48-3.8) | 0.77(0.25-2.36) | 1.47(0.53-4.11) | 1.07(0.42-2.77) | 0.58(0.17-1.91) |
| Niacin | 4.49(2.06-9.77)** | 2.25(0.86-5.86) | 1.29(0.50-3.30) | 4.15(1.95-8.83)** | 2.25(0.84-6.06) | 1.34(0.62-2.91) | 3.60(1.97-6.57)** | 1.73(0.72-4.17) | 1.23(0.48-3.17) |
| RBC folate | 0.93(0.64-1.36) | 0.93(0.57-1.50) | 0.96(0.62-1.48) | 0.94(0.64-1.37) | 0.93(0.56-1.56) | 0.96(0.60-1.53) | 0.93(0.61-1.40) | 0.93(0.54-1.58) | 0.95(0.55-1.63) |
| Q1, quantiles 1; Q2, quantiles 2; Q3, quantiles 3; Q4 quantiles 4;  OR (95% CI), odds ratio (95% confidence interval).  *P < 0.05, **P < 0.01.  DFE, dietary folate equivalents.  Model 1 was adjusted for age.group, weight.group, and race/ethnicity.  Model 2 was adjusted for covariates in model 1, and also education, physical activity, smoking.  Model 3 was adjusted for covariates in model 2, and also hypertension, diabetes, and dietary intakes of cholesterol and hyperuricemia, while eliminate weigh.group because of collinearity | | | | | | | | | |
