# Supplementary material for: Association between dietary intakes of B vitamins and nonalcoholic fatty liver disease in postmenopausal women: a cross-sectional study
Source: Front Nutr. 2023 Oct 19;10:1272321. doi: 10.3389/fnut.2023.1272321 (PMC10621796; doi:10.3389/fnut.2023.1272321)
Supplement: Supplementary file 3 [file Table_2.DOCX]

**Support table 2**. **Associations between B vitamin and liver fibrosis in postmenopausal women(including in non-Hispanic white, non-Hispanic black, and Mexican-American).**

| **Exposure** | Model 1 Model 2 Model 3 | | | | | |
| --- | --- | --- | --- | --- | --- | --- |
|  | **T2 vs. T1**  **OR (95%CI)** | **T3 vs. T1**  **OR (95%CI)** | **T2 vs. T1**  **OR (95%CI)** | **T3 vs. T1**  **OR (95%CI)** | **T2 vs. Q1**  **OR (95%CI）** | **T3 vs. T1**  **OR (95%CI)** |
| Vitamin B1 | 1.13(0.48-2.63) | 0.94(0.38-2.32) | 1.14(0.48-2.71) | 0.86(0.34-2.19) | 1.06(0.39-2.88) | 1.00(0.37-2.73) |
| Vitamin B2 | 1.85(0.59-5.76) | 2.18(0.76-6.27) | 1.91(0.58-6.31) | 2.37(0.95-5.92) | 1.78(0.58-5.43) | 2.31(0.65-8.14) |
| Vitamin B6 | 0.95(0.25-3.54) | 1.44(0.46-4.49) | 0.93(0.19-4.54) | 1.48(0.48-4.55) | 0.92(0.26-3.23) | 1.43(0.42-4.87) |
| Vitamin B12 | 1.84(0.67-5.09) | 2.40(0.71-8.09) | 1.65(0.53-5.19) | 2.08(0.53-8.17) | 1.47(0.55-3.97) | 1.56(0.50-4.87) |
| Choline | 1.21(0.47-3.07) | 3.50(1.20-10.2)* | 1.11(0.44-2.81) | 3.41(0.93-12.6) | 0.72(0.20-2.60) | 1.80(0.34-9.49) |
| Folate, DFE | 1.24(0.48-3.19) | 0.76(0.25-2.25) | 1.35(0.55-3.30) | 0.70(0.21-2.35) | 1.22(0.48-3.11) | 0.72(0.22-3.29) |
| Niacin | 1.84(0.70-4.87) | 1.19(0.38-3.68) | 1.68(0.56-5.08) | 1.16(0.37-3.66) | 1.40(0.57-3.46) | 0.92(0.26-3.11) |
| RBC folate | 0.97(0.62-1.52) | 1.01(0.61-1.66) | 0.98(0.61-1.56) | 1.01(0.59-1.73) | 0.97(0.60-1.59) | 1.00(0.56-1.81) |
| T1, tertiles 1; T2, tertiles 2; T3, tertiles 3;  OR (95% CI), odds ratio (95% confidence interval).  *P < 0.05, **P < 0.01.  DFE, dietary folate equivalents.  Model 1 was adjusted for age.group, weight.group, and race/ethnicity.  Model 2 was adjusted for covariates in model 1, and also education, physical activity,smoke  Model 3 was adjusted for covariates in model 2, and also hypertension, diabetes, and dietary intakes of cholesterol and hyperuricemia,while eliminate smoke because of collinearity. | | | | | | |
