# Supplementary material for: Association between dietary intakes of B vitamins and nonalcoholic fatty liver disease in postmenopausal women: a cross-sectional study
Source: Front Nutr. 2023 Oct 19;10:1272321. doi: 10.3389/fnut.2023.1272321 (PMC10621796; doi:10.3389/fnut.2023.1272321)
Supplement: Supplementary file 4 [file Table_3.DOCX]

|  | Vitamin B1 | Vitamin B2 | Vitamin B6 | Vitamin B12 | Choline | Folate | Niacin | RBC folate |
| --- | --- | --- | --- | --- | --- | --- | --- | --- |
| **Age.group** | 0.60 | 0.70 | 0.60 | 0.4 | 0.40 | 0.60 | 0.40 | 0.80 |
| **Overweight** | 0.30 | 0.70 | 0.30 | 0.4 | 0.50 | 0.70 | 0.90 | 0.50 |
| **Race** | 0.20 | 0.053 | 0.20 | 0.052 | 0.40 | 0.40 | 0.20 | 0.20 |
| **Hypertention** | 0.60 | 0.40 | 0.80 | 0.9 | 0.70 | 0.90 | 0.60 | 0.30 |
| **Hyperuricemia** | 0.90 | 0.60 | 0.60 | 0.2 | 0.20 | 0.90 | 0.70 | 0.058 |
| **Diabetes** | 0.50 | 0.80 | 0.60 | 0.6 | 0.50 | 0.70 | 0.40 | 0.50 |
| **Exercise** | 0.40 | 0.90 | 0.13 | 0.073 | 0.30 | 0.60 | 0.40 | 0.30 |
| **Education** | 0.20 | 0.072 | 0.12 | 0.5 | 0.60 | 0.071 | 0.20 | 0.30 |
| **Cholesterol（mg/d）** | 0.089 | 0.063 | 0.061 | 0.052 | 0.50 | 0.70 | 0.040 | 0.070 |
| **chi-squared test with Rao & Scott’s second-order correction** | | | | | | | | |

**Support table 3**. **The p-value for the differences in covariates among the four groups divided based on quartiles in non-alcoholic fatty liver disease.**
